# From Tension to Compression: Asymmetric Mechanical Behaviour of Trabecular Bone’s Organic Phase

**DOI:** 10.1007/s10439-018-2009-7

**Published:** 2018-03-27

**Authors:** Shuqiao Xie, Robert J. Wallace, Anthony Callanan, Pankaj Pankaj

**Affiliations:** 1 School of Engineering, Institute for Bioengineering, The University of Edinburgh, Faraday Building, The King’s Buildings, Edinburgh, EH9 3DW UK; 2 Orthopaedics and Trauma, The University of Edinburgh, Chancellor’s Building, Edinburgh, EH16 4SB UK

**Keywords:** Demineralised bone, Inelastic buckling, Stiffness reduction, Bone volume ratio

## Abstract

Trabecular bone is a cellular composite material comprising primarily of mineral and organic phases with their content ratio known to change with age. Therefore, the contribution of bone constituents on bone’s mechanical behaviour, in tension and compression, at varying load levels and with changing porosity (which increases with age) is of great interest, but remains unknown. We investigated the mechanical response of demineralised bone by subjecting a set of bone samples to fully reversed cyclic tension–compression loads with varying magnitudes. We show that the tension to compression response of the organic phase of trabecular bone is asymmetric; it stiffens in tension and undergoes stiffness reduction in compression. Our results indicate that demineralised trabecular bone struts experience inelastic buckling under compression which causes irreversible damage, while irreversible strains due to microcracking are less visible in tension. We also identified that the values of this asymmetric mechanical response is associated to the original bone volume ratio (BV/TV).

## Introduction

With increasing ageing population, which is known to cause deteriorated bone quality, understanding the mechanical response of bone to loads has assumed increased importance. Bone is subjected to a wide range of loading regimes that include tension, compression and shear. Evaluation of the mechanical behaviour of bone has been the subject of numerous studies in which its elastic properties,^22^,^36^ its yielding and post elastic behaviour,^2^,^26^,^39^ its time dependent response to loading^8^,^29^,^30^,^49^ and its response to cyclic and fatigue loading^11^,^12^,^17^,^19^,^44^ have been considered. For trabecular bone, it is now recognised that its elastic moduli can be reasonably well predicted from the bone volume to total volume ratio (BV/TV) and indices of its microarchitecture such as mean intercept length and star volume distribution fabric tensors^36^,^43^,^50^; and its elastic limit in the strain space is fairly isotropic and largely independent of BV/TV.^3^,^26^ A few studies have used cyclic loading to understand the fatigue behaviour of cortical^11^,^12^,^17^ and trabecular bone,^19^,^44^ however apart from few studies,^11^,^12^ all others have been in either only in tension^17^ or only in compression.^19^,^44^ A few trabecular bone studies^19^,^44^ have also successfully related its strain response under cyclic loading to indices of bone micro-structure.

Bone is a composite material which comprises of a mineral phase (mainly carbonated hydroxyapatite), organic phase (mostly type I collagen) and water assembled into a complex, hierarchical structure.^16^,^48^ Mechanically collagen and mineral play very different roles—the elastic modulus of collagen is much lower than that of the hydroxyapatite, but the former is three orders of magnitude tougher.^38^ While the mineral provides the stiffness, it is much more brittle than the collagen. Therefore mineral-collagen ratio (known to increase with age in humans^1^) has a role in the mechanical behaviour of trabecular bone. For example, evaluation of elastic modulus of bone from different species has shown that it increases with mineral content.^18^ Tests conducted on demineralised compact bovine humeral diaphyseal bone samples show that they had an elastic modulus of around 600 MPa^9^; untreated cortical bone (extracted samples without any chemical treatment) on the other hand is reported to have an elastic modulus in excess of 6 GPa.^18^ Study of the mechanical behaviour of bone’s constituents, therefore, is important in several contexts. Firstly the mechanical behaviour of the constituents helps in the understanding of the bone behaviour as a composite.^9^,^20^,^28^ This in conjunction with changing mineral collagen ratio and porosity with age gains significant importance.^28^ The effect of the contribution of bone constituents is not only on its elastic modulus^9^ but also on its strength,^15^ and its behaviour under cyclic loads.^35^ Secondly, it has been suggested that data on the mechanical properties of collagen has clinical relevance in the early stages of fracture repair before bone mineralisation occurs.^9^ Thirdly, though not considered in this study, the two main constituents of bone are known to play very different roles in its time-dependent behaviour.^7^

To evaluate the mechanical behaviour of the organic phase, a number of previous studies have undertaken mechanical tests on demineralised bone; almost all of which have been on cortical bone.^7^,^9^,^10^,^14^,^34^,^35^ Evaluation of elastic modulus and strength through monotonically increasing loading in tension^9^,^10^,^14^ or compression^34^ has been the focus of most studies. Bone and consequently its constituents are subjected to cyclic loading.^44^ Novitskaya *et al*., conducted cyclic loading tests on demineralised cortical bone in three different directions and showed that cortical bone has anisotropic cyclic behaviour with larger energy dissipation in transverse directions.^35^ Loading cycles in this cited study were confined to compression although the contribution of the organic phase to tension has been noted to be much more significant.^10^ Studies conducted on the mechanical behaviour of demineralised trabecular bone are limited, confined to monotonic loading in compression and generally conducted with an aim to evaluate elastic modulus and strength.^15^ These limited studies indicate that there is considerable gap in understanding the mechanical behaviour of the organic phase of bone under cyclic loading at different load levels.

This study aims to analyse the mechanical behaviour of demineralised trabecular bone in tension and compression using a novel experimental protocol. Firstly it aims to evaluate the response due to fully reversed cyclic loading to examine how samples behave in tension and compression. Such tests have not been previously conducted for demineralised bone and are rare even for untreated bone.^11^,^12^ Secondly it aims to evaluate how the cyclic response varies with application of different load levels. Few tests conducted on untreated bone (and limited to compression) have shown that the response varies with load level.^44^ Lastly by undertaking a micro-CT (*µ*CT) of samples prior to demineralisation this study aims to consider how the response is influenced by the original BV/TV of trabecular bone. Our hypothesis is that the mechanical behaviour of demineralised trabecular bone has tension compression asymmetry, varies with load levels and is associated with its porosity.

## Materials and methods

Fresh proximal tibia, from bovine (under 30 months old when slaughtered), were obtained from a local abattoir and stored at − 20 °C until utilised. The bones were allowed to thaw at room temperature before bone cores were extracted along its principal axis, using diamond coring tools (Starlite, Rosement, IL, USA). A low speed rotating saw (Buehler, Germany) was used to create parallel sections and to trim growth plates if they were present. All coring and cutting were conducted in a water bath to avoid excessive heat generation. The cylindrical bone samples (*n* = 5) had a diameter of 10.6  ± 0.1 mm and mean height of 22.1 ± 0.7 mm.

Bone marrow was removed from each sample using a dental water jet (Interplak, Conair) with tap water at room temperature.^27^ All the samples were then centrifuged at 2000 r.p.m for 2 h to remove any residual marrow.^41^ All the samples were scanned using *µ*CT scanner (Skyscan 1172, Bruker, Kontich, Belgium) at a resolution of 17.22 *µ*m and the system’s software was used to evaluate bone volume to total volume ratio (BV/TV) of the bone, which was found to be in the range 21–32%. Scanning parameters used were: source voltage 54 kV, current 185 *μ*A, exposure 885 ms with a 0.5 mm aluminium filter between X-ray source and the sample. The image quality was improved by using two frames averaging.

After scanning, demineralisation was conducted by submerging samples in 20 ml 0.6 N hydrochloric acid (HCl) at room temperature assisted by a racking system. The solution was changed daily^31^ for 2 weeks after which the completeness of demineralisation was verified using *µ*CT scanning. All samples in this study were found to be fully demineralised in 2 weeks. It should be noted that although EDTA solution has been previously used to demineralise bone, we used HCl because the process is much quicker and has been employed successfully in previous studies.^10^,^13^,^15^,^16^

**Figure 1:**
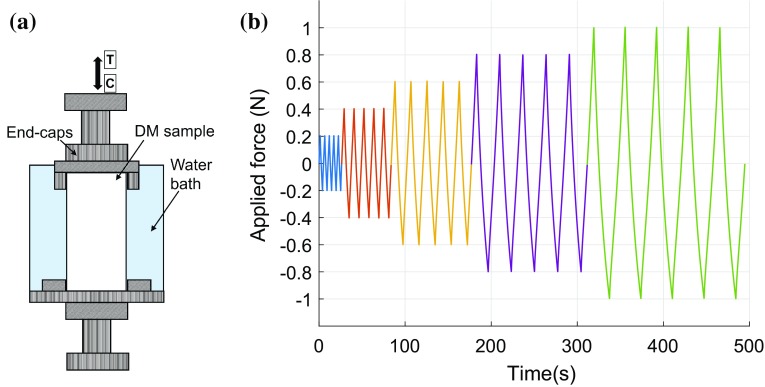
Experiment set up and load application. Schematic diagram of the test sample set up (**a**) and loading cycles applied (**b**).

Samples were fixed into end-caps (Fig. 1a) using bone cement (Simplex, Stryker, UK) with the assistance of a custom made alignment tool in order to minimise end-artefacts during testing.^23^ The effective length (19.1 ± 0.7 mm) of each sample was calculated as the length of the sample between the end-caps plus half the length of the sample embedded within the end-caps.^23^ Each sample was placed in an epoxy tube filled with PBS to ensure that they remained hydrated at all stages of mechanical testing.

Each sample was subjected to reversible cyclic loading by means of an Instron material testing machine (50 N load cell, Model 3367). Samples were subjected to 5 loading cycles varying from tension to compression to the same axial force amplitude after which the load level was increased (Fig. 1b). Five load levels were selected: 0.2, 0.4, 0.6, 0.8 and 1.0 N (corresponding to average axial stress varying from 2.27 to 11.33 kPa). The choice of 5 cycles at each load level was based on preliminary tests, which showed that most variation in strain (or displacement) response occurred in the first five cycles, after which this variation was very small. Our preliminary tests also showed that initiating the first cycle in tension or compression made little difference to the strain response. Cyclic loads were applied under strain control, i.e., the strain was slowly increased till the required load level was achieved. A very slow 0.1%/s strain-based loading rate was used to minimise the heat generation (e.g., demineralised samples took from 1 to 11 s to attain a load of 0.2 N).

## Results

**Figure 2:**
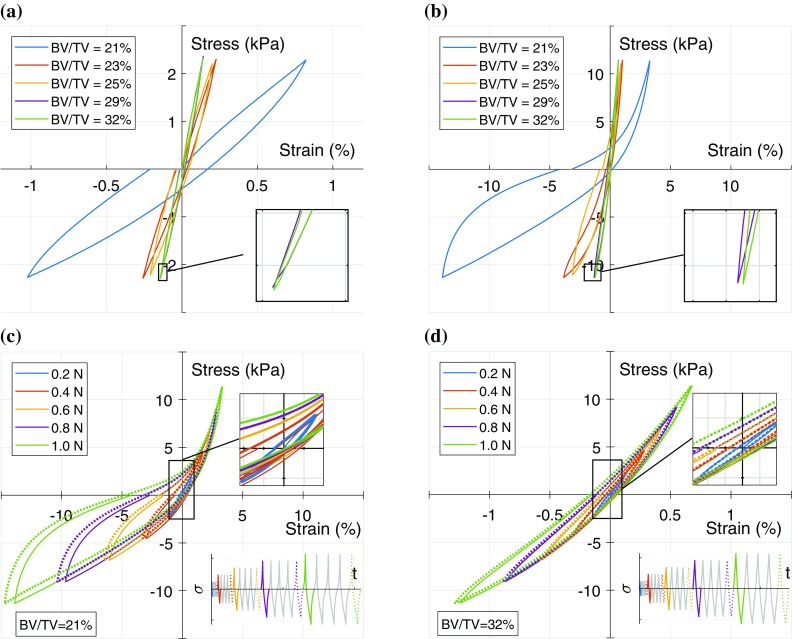
The stress–strain loops for demineralised trabecular bone samples under fully reversed tension–compression cyclic loading. Curve for samples tested at load level 1 (0.2 N) (**a**) and load level 5 (1 N) (**b**) for the first cycle of loading. Comparison of all load levels for samples with BV/TV = 21% (**c**) and BV/TV = 32% (**d**). For clarity only the response to the first (solid line) and the fifth (dotted line) loading cycles are shown for each load level. Inset shows load application.

The stress–strain curves for the first cycle at the lowest load level (0.2 N) and the highest load level (1.0 N) are shown in Figs. 2a and 2b, respectively. It is apparent that the resulting strain response is associated with the sample’s original BV/TV; samples with higher BV/TV experience much lower strain in comparison to the more porous samples. For example, the sample with BV/TV = 32% experienced only 0.14% strain in tension compared to 0.82% strain observed for sample with BV/TV = 21% (Fig. 2a). Comparing tension (taken as positive) and compression for the first load cycle, it can be seen that the differences in axial strain magnitude is small for samples with higher BV/TV and the difference increases with increasing porosity and with increasing load level (Fig. 2b). It is clear that the mechanical behaviour of demineralised trabecular bone is strongly dependent on its original BV/TV.

This trend is consistent for all cycles and at all load levels. This is illustrated in Figs. 2c and 2d which show the cyclic loading history for two typical samples; the insets show load application. For clarity, only the first and fifth cycles for each load level are shown. Comparing Figs. 2c and 2d, it is apparent that the higher BV/TV sample experiences lower strains at the same load level (i.e., it is stiffer) for both tension and compression. Nonlinearity of the stress–strain response is also more pronounced for the lower BV/TV sample. This BV/TV dependence was observed with all the samples.

Another apparent observation from the shape of the curves in Fig. 2 is that the demineralised samples become stiffer with increasing stress in tension and exhibit stiffness reduction with increasing stress in compression; this was observed at all load levels, in all cycles and for all tested samples. More importantly, the transition from tension to compression is smooth for every load level (Figs. 2c and 2d); this was observed for all the samples tested. Further examination of the loading and unloading curves in compression indicates that buckling is not entirely elastic. Figure 2 clearly indicates that the original BV/TV plays an important role in the cyclic response of demineralised trabecular bone. It is important to note that all samples were extracted in the same direction, from similar anatomical site, from cattle of about the same age and employing the same demineralisation process i.e. by using HCl solution.

**Figure 3:**
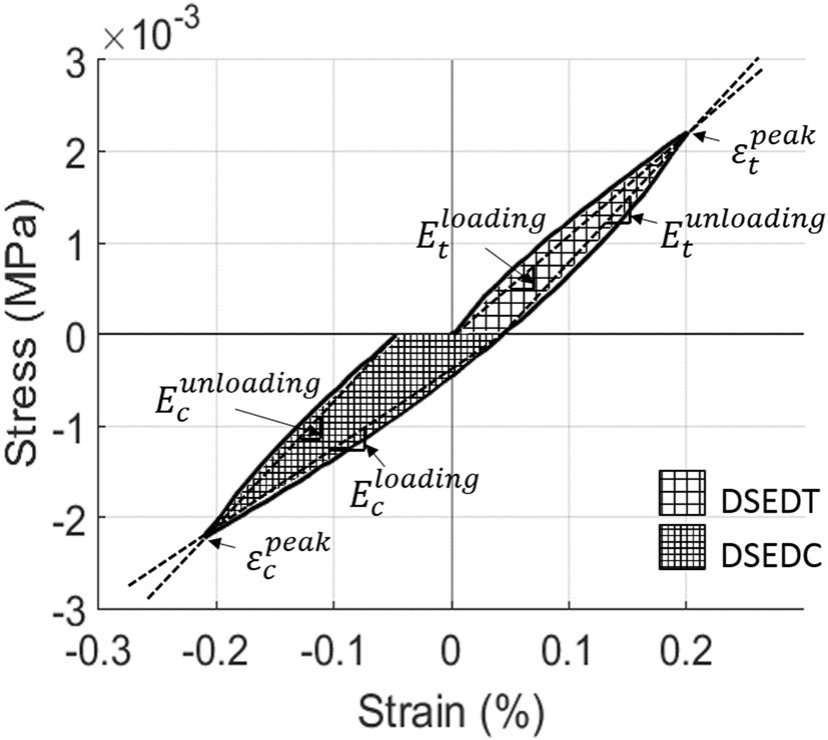
Definition of terms: $\varepsilon_{\text{t}}^{\text{peak}}$ and $\varepsilon_{\text{c}}^{\text{peak}}$ are the peak strains at the end of a loading cycle in tension and compression respectively. The secant moduli represent four different slopes for one complete cycle with subscripts ‘t’ and ‘c’ denoting tension and compression respectively, and the superscripts representing loading and unloading branches. DSEDT and DSEDC represent dissipated strain energy in tension and compression, respectively.

To further evaluate the cyclic response we examined ratcheting strain and dissipated strain energy density in tension (DSEDT) and compression (DSEDC), as shown in Fig. 3, for all load levels and for all samples. We also considered the secant moduli, defined as four different slopes for one complete cycle of loading and unloading in tension and compression as shown in Fig. 3. Ratcheting strain can be defined as the average of peak strain in tension $\left( {\varepsilon_{\text{t}}^{\text{peak}} } \right)$ taken as positive and compression $\left( {\varepsilon_{\text{c}}^{\text{peak}} } \right)$ taken as negative at the same load level (Fig. 3). A non-zero ratcheting strain only occurs when the mechanical properties in tension and compression are different.^40^

**Figure 4:**
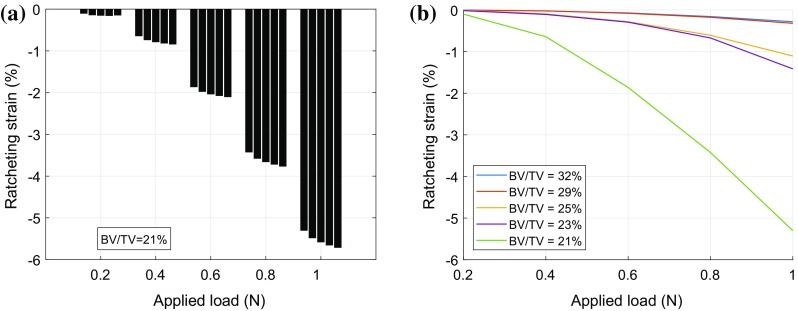
Ratcheting strain $\left( {{{\left( {\varepsilon_{\text{t}}^{\text{peak}} + \varepsilon_{\text{c}}^{\text{peak}} } \right)} \mathord{\left/ {\vphantom {{\left( {\varepsilon_{\text{t}}^{\text{peak}} + \varepsilon_{\text{c}}^{\text{peak}} } \right)} 2}} \right. \kern-0pt} 2}} \right)$ for one typical sample (**a**), and with variation in BV/TV (**b**). (**a**) Ratcheting strain in each load cycle for BV/TV = 21%, (**b)** Ratcheting strain for all samples in first load cycle.

We first considered the most porous sample (with BV/TV of 21%) for demonstrating the variation in ratcheting strain (*ɛ*_*r*_) in different cycles and at different load levels (Fig. 4a). Ratcheting strain was consistently negative, which implies that the demineralised samples experience larger strain in compression than in tension at the same load level (Fig. 4a). Even for this most porous sample the ratcheting strain only increases marginally with increasing cycle number; while the increase with load level is nonlinear and much more significant. Next we considered the ratcheting strain for all five samples in the first load cycle for all load levels. As expected, the magnitude of ratcheting strains is much larger for samples with lower BV/TV (Fig. 4b). Also the ratcheting strains are consistently negative and their magnitude increases rapidly with load level (Fig. 4) indicating that the organic phase has a much better load bearing capability in tension without significant additional strains than in compression.

**Figure 5:**
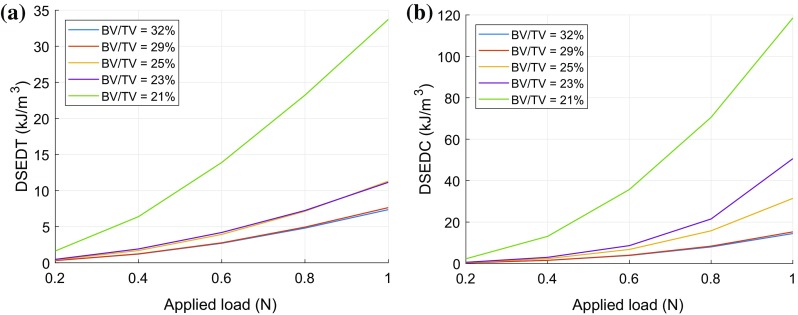
Dissipated strain energy density in tension and compression with varying load levels for all 5 samples. DSEDT (**a**) and DSEDC (**b**).

DSEDT and DSEDC were calculated by integrating corresponding areas, as discussed and the results are shown in Fig. 5 for the first cycle for each load level. Both DSEDT and DSEDC increase with increasing load levels, and dissipated strain energy values and their rate of change increases with decreasing BV/TV (Fig. 5). Energy dissipation in compression was found to be consistently higher than in tension. This is because the samples experience not only lower strains in tension but also because tensile strains do not have large irreversible component. On the other hand, in compression the samples experience large strains and these include relatively large irreversible strains due to inelastic buckling of collagen struts.

**Figure 6:**
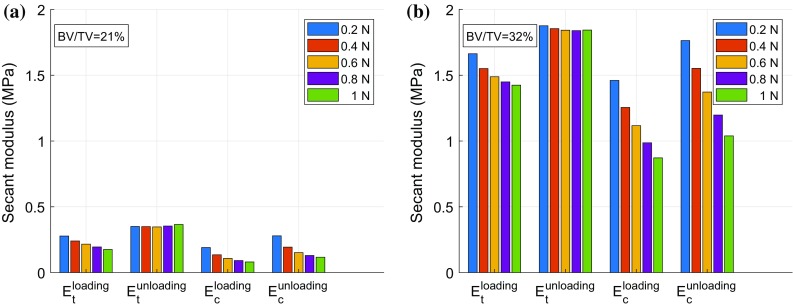
Secant moduli for two samples. BV/TV = 21% (**a**) and BV/TV = 32% (**b**).

As discussed, four secant moduli were evaluated (Fig. 6): $E_{\text{t}}^{\text{loading}}$, $E_{\text{t}}^{\text{unloading}}$, $E_{\text{c}}^{\text{loading}}$ and $E_{\text{c}}^{\text{unloading}}$. These are illustrated for samples with the largest and smallest BV/TV in Fig. 6. As expected, the porous sample has smaller secant moduli in comparison to the denser sample (i.e. $E_{\text{t}}^{\text{loading}} = 0.28\;{\text{MPa}}$ for BV/TV = 21% compared with 1.66 MPa for BV/TV = 32%). The unloading modulus is always higher than the loading modulus in both tension and compression. With increasing load level $E_{\text{t}}^{\text{unloading}}$ remains almost constant, while $E_{\text{t}}^{\text{loading}}$ decreases slightly. In contrast to tension, $E_{\text{c}}^{\text{loading}}$ and $E_{\text{c}}^{\text{unloading}}$ both decrease dramatically with increasing load level. This interesting trend, followed by all the samples, indicates that while compression leads to significant irreversible strain with increasing load in the demineralised microstructure of bone, this is relatively small in tension.

## Discussion

This study considered fully reversible tension–compression cyclic loading tests on five demineralised trabecular bone samples with BV/TV ranging from 21 to 32%. Samples were subjected to five different load levels (0.2 to 1 N at 0.2 N interval denoted as load level 1–5), and five cycles were applied at each load level. The asymmetric responses of the organic phase of trabecular bone were found when it loaded cyclically from tension to compression. The study shows demineralised trabecular microstructure stiffens in tension and undergoes stiffness reduction in compression. The trend of the asymmetric mechanical response is associated to the original BV/TV.

In previous studies the shape of loading curve in compression for untreated trabecular bone have shown a reduced load carrying capacity with increasing load^21^,^25^,^32^ but unloading demonstrates that much of this is due to irrecoverable plastic strain.^24^,^33^,^37^ It is perhaps not improper to infer that this is due to the damage and failure of the mineral phase. Stiffness reduction in compression has been previously observed for demineralised cortical bone.^35^ For trabecular bone, however, the stiffness reduction is likely to be accentuated due to elastic and inelastic buckling of demineralised trabecular struts. Previous tests in tension on demineralised cortical bone have shown stiffening with load increase,^9^,^14^ similar to what was observed in this study. For untreated bone, however, it is stiffness reduction (rather than stiffness increase) that has been previously observed in tension as well,^25^ which can be attributed to failure of the mineral-collagen interface.

To the best of our knowledge, there have been no previous tests on demineralised bone, cortical or trabecular that have considered fully reversible cyclic loading. However, similar compression-softening and stretch-stiffening have been previously observed in semi-flexible biopolymers^45^ where it has been suggested that this asymmetric response in tension and compression is caused by the bending and/or buckling stress in fibres under compression, force for which is much lower than the load required for straightening and stretching. This behaviour is akin to a rectangular steel frame braced along one diagonal and subjected to shear.^5^ When the diagonal brace is in tension the deformation of the frame is limited but when it is in compression its inelastic buckling results in larger deformation and residual deformations.

Observed irreversible strain, we believe, is due to inelastic buckling in compression as stated above and has implications for old/osteoporotic bone. Ageing bone not only leads to reduction in BV/TV but also relative increase in mineral to collagen ratio.^1^ Consequently there is increased reliance on the limited organic phase to provide ductility; our tests show that the demand to sustain increasing magnitudes of strain by the organic phase increases dramatically in compression with decreasing BV/TV. At the macro scale the behaviour of bone in tension and its fracture toughness have been seen as key to bone fracture^46^ while our study indicates that possible failure due to inelastic trabecular buckling in compression needs greater consideration. Mineral deposition increases the elastic modulus of bone and hence the buckling load, however once bucking is initiated (which is more likely in low BV/TV bone) then it is likely to be inelastic due to limited contribution of mineral in tension. Buckling has been previously proposed as the probable cause of failure for vertebral trabecular bone.^4^,^42^ Our study demonstrated that this buckling of trabeculae could be initiated from organic phase of trabecular bone. This study also shows that the possibility of hip fractures in the elderly occurring due to normal physiological activities, such as level walking, resulting in the individual falling down (rather than the fracture being caused by a fall) does exist.^47^

A few studies have attempted to develop predictive models of the mechanical behaviour of bone based on the properties of the mineral, organic phase and their interaction at either the solid phase level^20^ or in terms of demineralised and deproteinised macro level.^28^ These cited studies have been limited to the prediction of elastic properties and have not distinguished between compression and tension. Our study can help take these predictive models forward. It is important to note that many of the findings in this study were only made possible by the novel experimental protocol which permitted evaluation of demineralised samples at different load levels and in both tension and compression.

Our work suffers from a number of limitations. Firstly, all the tests were conducted at room temperature; creep behaviour has been reported to be temperature dependent.^6^ Secondly, the stress–strain responses were measured directly from the machine rather than using extensometer, but the aim of the paper is to compare the trends—response to reversible cyclic loading from tension to compression and across samples with different BV/TV prior to demineralisation. Lastly, since we only considered a limited number of samples, a statistical analysis that considers the influence of different variables was not possible. These trends, we believe, are real despite the limitations.

This study, we believe, makes several important contributions. Firstly it develops a novel experimental protocol that can evaluate the mechanical response of materials under cyclic loads that range from tension to compression and are of varying magnitudes. The study will help in the development of composite models from the mechanical response of its constituents. We have shown that the behaviour of the organic phase of trabecular bone has tension–compression asymmetry and varies with load levels and porosity. Interestingly, the transition from tension to compression is found to be smooth for all load levels. Collagen struts stiffen in tension while they undergo inelastic bucking in compression. These findings may explain, at least partially, the reasons for non-traumatic fractures in the elderly as increasing bone porosity and reduced collagen to mineral ratio will result in higher risk of buckling failure.
